# Supplementary material for: Optimizing High-Efficiency Quantum Memory with Quantum Machine Learning for Near-Term Quantum Devices
Source: Sci Rep. 2020 Jan 10;10:135. doi: 10.1038/s41598-019-56689-0 (PMC6954268; doi:10.1038/s41598-019-56689-0)
Supplement: Supplementary file 1 — Supplemental Information. [file 41598_2019_56689_MOESM1_ESM.pdf]

Optimizing High-Efficiency Quantum Memory with Quantum Machine Learning for Near-Term Quantum Devices

Laszlo Gyongyosi[1,2,3,*] **and Sandor Imre**[2]

[1]School of Electronics and Computer Science, University of Southampton, Southampton, SO17 1BJ, UK
[2]Department of Networked Systems and Services, Budapest University of Technology and Economics, Budapest, H-1117 Hungary
[3]MTA-BME Information Systems Research Group, Hungarian Academy of Sciences, Budapest, H-1051 Hungary
[*]l.gyongyosi@soton.ac.uk

ABSTRACT

Quantum memories are a fundamental of any global-scale quantum Internet and high-performance quantum networking. A main problem of quantum memories is the low retrieval efficiency of the quantum systems from the quantum registers of the quantum memory. Here, we define a novel quantum memory called high-retrieval-efficiency (HRE) quantum memory for near-term quantum devices. An HRE quantum memory unit integrates local unitary operations on its hardware level for the optimization of the readout procedure and utilizes the advanced techniques of quantum machine learning. We define the integrated unitary operations of an HRE quantum memory, prove the learning procedure, and evaluate the achievable output signal-to-noise ratio values. We prove that the local unitaries of an HRE quantum memory achieve the optimization of the readout procedure in an unsupervised manner without the use of any labeled data or training sequences. We show that the readout procedure of an HRE quantum memory is realized in a completely blind manner without any information about the input quantum system or about the unknown quantum operation of the quantum register. We evaluate the retrieval efficiency of an HRE quantum memory and the output SNR (signal-to-noise ratio). The results are particularly convenient for gate-model quantum computations and the near-term quantum devices the quantum Internet.

A Appendix

A.1 Abbreviations

DFT Discrete Fourier Transform

DSTFT Discrete Short-Time Fourier Transform

HRE High-Retrieval Efficiency

SNR Signal-to-Noise Ratio

A.2 Notations

The notations of the manuscript are summarized in Table A.1.

Table A.1. Summary of notations.

Notation	*Description*
ρ_{in}	An unknown input quantum system formulated by n unknown density matrices.
ρ_i	An i-th density matrix, $i = 1, \ldots, n$.
QR	Quantum register of an HRE quantum memory.
σ_{QR}	Mixed state of the QR quantum register.
T	Total evolution time, $t = 1, \ldots, T$.
$\sigma_{QR}^{(t)}$	Mixed state of the QR quantum register at a given t, $\sigma_{QR}^{(t)} \in \mathbb{C}$, $\sigma_{QR}^{(t)} = \sum_{i=1}^{n} \lambda_i^{(t)} \left\| \varphi_i^{(t)} \right\rangle \left\langle \varphi_i^{(t)} \right\|$, $t = 1, \ldots, T$.
$U_{QR}^{(t)}$	Unknown evolution matrix of the QR quantum register at a given t.
$\dim\left(U_{QR}^{(t)}\right)$	Dimension of $U_{QR}^{(t)}$, $\dim\left(U_{QR}^{(t)}\right) = d^n \times d^n$, where d is the dimension of the quantum system.
$X_i^{(t)}$	A complex quantity, defined as $X_i^{(t)} = \sqrt{\lambda_i^{(t)}} \left\| \varphi_i^{(t)} \right\rangle$, $i = 1, \ldots, n$, $t = 1, \ldots, T$.
$X^{(t)}$	Sum of n complex quantities, $X^{(t)} = \sum_{i=1}^{n} X_i^{(t)}$.
$\zeta_{QR}^{(t)}$	An unknown residual density matrix at a given t, it formulates the mixed system of the quantum register as $\sigma_{QR}^{(t)} = \rho_{in} + \zeta_{QR}^{(t)}$.
M	Number of source systems of the mixed quantum register, $\sigma_{QR}^{(t)} = \sum_{m=1}^{M} \rho_m$, where ρ_m is an m-th source system, $m = 1, \ldots, M$.
ρ_m	An m-th source density matrix of the mixed quantum register state, $m = 1, \ldots, M$.
$X_i^{(m,t)}$	A complex quantity associated with an m-th source system, $X_i^{(m,t)} = \sqrt{\lambda_i^{(m,t)}} \left\| \varphi_i^{(m,t)} \right\rangle$, $m = 1, \ldots, M$, $i = 1, \ldots, n$, $t = 1, \ldots, T$.
$X^{(m,t)}$	Sum of n complex quantities, $X^{(m,t)} = \sum_{i=1}^{n} X_i^{(m,t)}$, $m = 1, \ldots, M$, $t = 1, \ldots, T$.
$\tilde{X}^{(m,t)}$	An approximation of $X^{(m,t)}$.
U_{QR}	Unknown transformation matrix of the QR quantum register over the total evolution time T.
V_{QG}	Inverse matrix of the unknown U_{QR}.
σ_{out}	Output quantum system.
U_{ML}	Unitary of a quantum machine learning procedure.

U_F	Factorization unitary, evaluates K bases for the source system decomposition, and defines a W auxiliary quantum system.
U_{CQT}	Unitary of the quantum constant Q transform. The U_{CQT} transform is a preliminary operation for the partitioning of the K bases onto M clusters via unitary U_P.
f_W	A windowing function in U_{CQT}.
U_P	A basis partitioning unitary that clusters the bases with respect to the M source systems.
$U_{CQT}^{\dagger}$	A unitary, inverse of U_{CQT}.
$\tilde{U}_{\text{DSTFT}}^{\dagger}$	Unitary of the inverse quantum DSTFT (discrete short-time Fourier transform) operation.
U_{DFT}	Quantum discrete Fourier transform.
u_{mk}	Parameter in the basis estimation procedure of U_{ML}, $m = 1, \ldots, M$, $k = 1, \ldots, K$.
H_{mk}	A Hamiltonian, $H_{mk} = G_{mk} \vert k_m \rangle \langle k_m \vert$, where G_{mk} is the eigenvalue of basis $\vert k_m \rangle$, $H_{mk} \vert k_m \rangle = G_{mk} \vert k_m \rangle$.
τ	Application time, sub-parameter of u_{mk}.
w_{kt}	A system state evolved via U_{ML}.
K	Number of bases evolved via U_{ML}.
T	Total evolution time of the quantum system in the quantum register.
M	Number of source systems of the mixed quantum system σ_{QR} of the quantum register.
U_B	A complex basis matrix, $U_B = \{u_{mk}\} \in \mathbb{C}^{M \times K}$, $m = 1, \ldots, M$, $k = 1, \ldots, K$.
$\vec{\rho}_W$	A complex matrix, $\vec{\rho}_W \in \mathbb{C}^{K \times T}$, $\vec{\rho}_W = \left\{ \rho_W^{(t)} \right\}_{t=1}^{T}$.
$W_k^{(t)}$	A complex quantity, $W_k^{(t)} \in \mathbb{C}$, $W_k^{(t)} = \sqrt{v_k^{(t)}} \vert \phi_k \rangle$, $m = 1, \ldots, M$, $k = 1, \ldots, K$.
w_{kt}	A complex quantity, $w_{kt} = W_k^{(t)}$, $m = 1, \ldots, M$, $k = 1, \ldots, K$.
W	A complex matrix, $W = \left\{ W_k^{(t)} = w_{kt} \right\} \in \mathbb{C}^{K \times T}$.
$\vec{X}$	A complex matrix, $\vec{X} \in \mathbb{C}^{M \times T}$.
$\tilde{X}$	A complex matrix, $\tilde{X} \in \mathbb{C}^{M \times T}$, an approximation of $\vec{X}$, as $\tilde{X} = U_B W$.
K_m	The number of bases associated with the m-th source system, $m = 1, \ldots, M$, $\sum_m K_m = K$.
$\vert \Phi^* \rangle$	Target output system state, $\vert \Phi^* \rangle = \frac{1}{\sqrt{K_1}} \sum_{k_1=1}^{K_1} \vert k_1 \rangle$, where K_1 is the number of bases for the source system $m = 1$, $k_1 = 1, \ldots, K_1$.
$\rho_{\vec{X}}$	A density matrix associated with $\vec{X}$, $\rho_{\vec{X}} = \sum_{m=1}^{M} \sum_{t=1}^{T} \vec{X}^{(m,t)} \left(\vec{X}^{(m,t)} \right)^{\dagger}$.
$\rho_{\tilde{X}}$	A density matrix associated with $\tilde{X}$, $\rho_{\tilde{X}} = \sum_{m=1}^{M} \sum_{t=1}^{T} \tilde{X}^{(m,t)} \left(\tilde{X}^{(m,t)} \right)^{\dagger}$.
$D(\cdot \Vert \cdot)$	Quantum relative entropy function.
$f(U_F)$	Objective function of unitary U_F.
$\mathscr{L}(\cdot)$	A likelihood function.
α_{mk}	A control parameter defined for u_{mk}, such that $u_{mk} \simeq \alpha_{mk} e^{-\alpha_{mk} u_{mk}}$.
β_{kt}	A control parameter defined for w_{kt}, such that $w_{kt} \simeq \beta_{kt} e^{-\beta_{kt} w_{kt}}$.

ζ	A set of model parameters, $\zeta = \{U_B, W\}$.
$\tau_{mk}^{(t)}$	A set of control parameters, $\tau_{mk}^{(t)} = \{\alpha_{mk}, \beta_{kt}\}$.
$\tilde{\zeta}$	A maximum likelihood estimation of ζ.
$\mathscr{D}(\cdot)$	A probability distribution.
κ_{mkt}	An estimation coefficient, $\kappa_{mkt} = u_{mk} w_{kt}$.
$\vec{\kappa}$	A complex matrix, $\vec{\kappa} \in \mathbb{C}^{M \times T}$, $\vec{\kappa} = \left\{\kappa^{(1,t)}, \ldots, \kappa^{(M,t)}\right\}_{t=1}^{T}$, where $\kappa^{(m,t)} = \left(\kappa_{k=1}^{(m,t)}, \ldots, \kappa_{k=K}^{(m,t)}\right)^T$, with $\kappa_k^{(m,t)} = \kappa_{mkt}$.
$\mathscr{D}_v(\cdot)$	A variational distribution.
$H(\mathscr{D}_v(\cdot))$	Entropy of a variational distribution $\mathscr{D}_v(\cdot)$.
$\mathscr{L}_{\mathscr{D}_v}$	A likelihood function.
$\mathbb{E}_{\mathscr{D}_v(i \neq \Phi)}(\cdot)$	Expectation function of the $\mathscr{D}_v(i)$ variational distribution of i, such that $i \neq \Phi$, $\Phi \in \{\vec{\kappa}, U_B, W\}$,<br>$\mathbb{E}_a(f(a) + g(a)) = \mathbb{E}_a(f(a)) + \mathbb{E}_a(g(a))$,<br>for some functions $f(a)$ and $g(a)$, and<br>$\mathbb{E}_a(bf(a)) = b\mathbb{E}_a(f(a))$<br>for some constant b.
$f_\delta(\cdot)$	Dirac delta function.
$f_\Gamma(\cdot)$	Gamma function,<br>$f_\Gamma(x) = \int_0^\infty t^{x-1} e^{-t} dt$.
$\mathscr{M}$	A multinomial distribution.
η_{mkt}	A multinomial parameter.
$\eta_k^{(m,t)}$	A multinomial parameter vector, $\eta_k^{(m,t)} = \left(\eta_{k=1}^{(m,t)}, \ldots, \eta_{k=K}^{(m,t)}\right)^T$,<br>$\sum_{k=1}^{K} \eta_k^{(m,t)} = 1$.
$\mathscr{G}(\cdot)$	A Gamma distribution,<br>$\mathscr{G}(x; a, b) = e^{(a-1)\log x - \frac{x}{b} - \log f_\Gamma(a) - a \log b}$,<br>where a is a shape parameter, while b is a scale parameter.
$f_\Gamma(\cdot)$	Gamma function.
$H(\mathscr{G}(\cdot))$	Entropy of Gamma distribution $\mathscr{G}(\cdot)$.
$\partial_{\mathscr{G}_{\log}}(\cdot)$	Derivative of the log gamma function (digamma function), $\partial_{\mathscr{G}_{\log}}(x) = \frac{d \log f_\Gamma(x)}{dx}$.
$\mathbb{E}(\kappa_{mkt})$	Expected value of κ_{mkt}, $\mathbb{E}(\kappa_{mkt}) = X^{(m,t)} \eta_{mkt}$.
$\tilde{\alpha}_{mk}(A)$	Control parameter for U_B, $\tilde{\alpha}_{mk}(A) = 1 + \sum_{t=1}^{T} \mathbb{E}(\kappa_{mkt})$.
$\tilde{\alpha}_{mk}(B)$	Control parameter for U_B, $\tilde{\alpha}_{mk}(B) = \frac{1}{\sum_{t=1}^{T} \mathbb{E}(w_{kt}) + \alpha_{mk}}$.
$\tilde{\beta}_{kt}(A)$	Control parameter for W, $\tilde{\beta}_{kt}(A) = 1 + \sum_{m=1}^{M} \mathbb{E}(\kappa_{mkt})$.
$\tilde{\beta}_{kt}(B)$	Control parameter for W, $\tilde{\beta}_{kt}(B) = \frac{1}{\sum_{m=1}^{M} \mathbb{E}(u_{mk}) + \beta_{kt}}$.
$\mathbb{E}(w_{kt})$	Expected value of $\mathbb{E}(w_{kt})$, $\mathbb{E}(w_{kt}) = \tilde{\beta}_{kt}(A)\, \tilde{\beta}_{kt}(B)$.
$\mathbb{E}(\log w_{kt})$	Expected value of $\mathbb{E}(\log w_{kt})$, $\mathbb{E}(\log w_{kt}) = \partial_{\mathscr{G}_{\log}}\left(\tilde{\beta}_{kt}(A)\right) + \log \tilde{\beta}_{kt}(B)$.
$\mathbb{E}(u_{mk})$	Expected value of $\mathbb{E}(u_{mk})$, $\mathbb{E}(u_{mk}) = \tilde{\alpha}_{mk}(A)\, \tilde{\alpha}_{mk}(B)$.

$\mathbb{E}(\log u_{mk})$	Expected value of $\mathbb{E}(\log u_{mk})$, $\mathbb{E}(\log u_{mk}) = \partial_{\mathscr{G}_{\log}}(\tilde{\alpha}_{mk}(A)) + \log \tilde{\alpha}_{mk}(B)$.
E_{mk}	A basis estimation, $E_{mk} \approx \alpha_{mk}$.
F_{kt}	A system state estimation, $F_{kt} \approx \beta_{kt}$.
$\tilde{\tau}_k^{(t)}$	Estimation of the control parameters α_{mk}, β_{kt}, as $\tilde{\tau}_{mk}^{(t)} = \{E_{mk}, F_{kt}\}$.
K^*	An optimal number of bases, $K^* = \arg\max_K \mathscr{L}_{\mathscr{D}_v}(K)$, where $\mathscr{L}_{\mathscr{D}_v}(K)$ is the likelihood function $\mathscr{L}_{\mathscr{D}_v}$ at a particular base number K.
Q	Parameter of U_{CQT}.
$f_W(\cdot)$	Windowing function for U_{CQT}.
h	Parameter of $f_W(\cdot)$.
C_B	A complex matrix of transformed bases, $C_B = U_{CQT}(U_B) = \{C_{mk}\} \in \mathbb{C}^{M \times K}$.
C_{mk}	A Q-transformed basis estimation parameter, $C_{mk} = U_{CQT}(E_{mk})$.
S	A complex matrix, $S = C_B W$, $S \in \mathbb{C}^{M \times T}$, $C_B W = (U_{CQT} U_B) W$.
$\mathscr{T}$	A tensor (multidimensional array).
$\dim(\mathscr{T})$	Dimension of tensor $\mathscr{T}$.
$\mathscr{R}$	A translation tensor.
$f(U_P)$	A cost function of U_P.
Ω	A sum of partitioned bases.
$\Omega_Q^{(m)}$	A cluster of $\left\|\Omega_Q^{(m)}\right\|$ Q-transformed bases for the m-th system state, $\Omega_Q^{(m)} = \left\{\Omega_Q^{(m,k_m)}\right\}_{k_m=1}^{K_m}$, $m = 1, \ldots, M$.
$\left\|\Omega_Q^{(m)}\right\|$	Cardinality of cluster $\Omega_Q^{(m)}$, $\left\|\Omega_Q^{(m)}\right\| = K_m$.
θ	Partitioned bases transformed by $U_{CQT}^{\dagger}$.
$\gamma^{(m)}$	A cluster of K_m bases for m-th system state.
χW	A system state, defined as $\chi W = U_{CQT}^{\dagger}(U_P(C_B W)) = U_{CQT}^{\dagger}(U_P U_{CQT} U_B)$.
$\|k_m\rangle$	A basis state associated with the m-th source.
x_{m,k_m}	Model parameter, defined as $x_{m,k_m} = \begin{cases} 0, \text{ if } m = 1 \\ !0, \text{ otherwise} \end{cases}$.
$\|\psi_{in}\rangle$	An arbitrary input system.
$\|\Phi^*\rangle$	An output system, $\|\Phi^*\rangle = U\|\varphi\rangle = U(U_{QR}\|\psi_{in}\rangle)$, where U is the operator of the integrated unitary operations of the HRE quantum memory, defined as $U = U_{ML}\tilde{U}_{\text{DSTFT}}^{\dagger} U_{DFT} = U_F U_{CQT} U_P U_{CQT}^{\dagger} \tilde{U}_{\text{DSTFT}}^{\dagger} U_{DFT}$.
$\mathscr{O}_V$	A verification oracle that computes the energy E of a wavefunction $\|\psi\rangle = \sum_i c_i \|\phi_i\rangle$.
$E(\psi)$	Energy E of a wavefunction $\|\psi\rangle = \sum_i c_i \|\phi_i\rangle$.
Δ	Wavefunction energy ratio difference, $\Delta = R(S,T) - R(S,X)$, where $R(S,T) = \frac{S}{T}$, $R(S,X) = \frac{S}{X}$, and $S = E(\psi_{in})$, $X = E(\varphi)$, and $T = E(\Phi^*)$.
Δ_{SNR}	An SNR difference.
